# Response to Canadian Neurophysiology Laboratory COVID-19 Practice Guidelines

**DOI:** 10.1017/cjn.2020.220

**Published:** 2021-01

**Authors:** Kenneth A. Myers

**Affiliations:** Research Institute of the McGill University Medical Centre, 1001 Décarie Blvd, Montreal, Quebec, Canada, H4A 3J1; Division of Neurology, Department of Pediatrics, Montreal Children’s Hospital, McGill University Health Centre, 1001 Décarie Blvd, Montreal, Quebec, Canada, H4A 3J1; Department of Neurology and Neurosurgery, Montreal Children’s Hospital, McGill University Health Centre, 1001 Décarie Blvd, Montreal, Quebec, Canada, H4A 3J1

I was pleased to see *CJNS* publish “Practice guidelines for Canadian neurophysiology laboratories during the COVID-19 pandemic,” a consensus guideline from the Canadian Society of Clinical Neurophysiologists.^1^ As the director of a neurophysiology laboratory, such guidelines are highly valuable in decision-making. While for the most part I agree with the recommendations, there are certain aspects that I feel bear review.

The authors did not include a Methods section, which is a standard practice in preparing clinical practice guidelines.^2^ When considering whether to follow a given guideline, a key factor is the rigor demonstrated in the literature review and process to reach consensus; however, in this case the authors only state in the Purpose that “Recommendations are based on expert opinion and review of relevant published guidelines.” One of the primary aims of the guidelines is reducing the risk of COVID-19 transmission to neurophysiology laboratory staff members, yet the author list does not note anyone with expertise in public health or infectious disease. If the authors had reached out for input from other specialties such as these, the guidelines would carry more weight.

Although most of the guidelines seem reasonable, I was struck by the statement “*Hyperventilation should not be routinely performed, but if justified for high diagnostic yield (e.g., paediatric absence epilepsy), patients must wear surgical masks*.” This is a blanket statement for practice during the pandemic, for all patients, whether suspected to have COVID-19 or not, regardless of the local burden of infection. As a pediatric epileptologist, for patients who can comply, hyperventilation has considerable utility in the majority of electroencephalograms (EEGs) I order, so this would represent a major change to our practice. There has, however, been anxiety amongst pediatric EEG technologists regarding infection transmission during hyperventilation, so the issue is not a trivial one and requires careful consideration.

Requiring patients to wear surgical masks will reduce the degree of hypocapnia achieved, and thus compromise the effectiveness of the provocative maneuver; other published COVID-19 literature has made the opposite recommendation, stating that hyperventilation should *never* be performed with a mask.^3–5^ I was curious as to what evidence supported the *CJNS* guideline, so I reviewed the three citations given by the authors to support the statement.

The authors cite three articles in support of their guideline on hyperventilation^6–8^; however, only one appears to even partially support their position. The article by Sethi is a short letter to the editor by a New York-based epileptologist describing his experiences adjusting laboratory practices during a period of overwhelming COVID-19 infections; there is no mention of hyperventilation. The paper by Haines et al comes from a single centre in Texas; the authors describe their experiences during the pandemic, and recommend that hyperventilation not be routinely performed in patients *with confirmed or presumed COVID-19 infection*, but make no comment as to changing routine EEG protocols for patients not at known risk for infection. Finally, the San-Juan et al. paper is a set of guidelines from the Latin Chapter of the International Federation of Clinical Neurophysiology; this very conservative document makes the somewhat confusing statement “Avoid maneuvers that promote airway handling. Avoid request of hyperventilation or use of CPAP” and cites “CDC, 2020” (I could not find a CDC statement commenting on hyperventilation).

Based on this review, the recommendation to wear a surgical mask during hyperventilation is not supported by any of the authors’ sources, and in fact other sources expressly recommend against this. The recommendation to change routine protocols to limit hyperventilation only to very high yield clinical scenarios (i.e., childhood absence epilepsy), was only endorsed by one of the three references given, and in that case no supporting evidence was given nor were there suggestions on what clinical scenarios would merit hyperventilation. While I understand the rush to publish quickly, this sort of misleading citation practice calls into question the reliability of the guidelines, particularly as the authors did not acknowledge other references providing very different guidance on the issue of hyperventilation, supported by accurate citations and rational argument.^3–5^

The decisions around hyperventilation and other neurophysiology laboratory protocols are complex, and should be based on several factors, including up-to-date local data on infection rates. For regions in which there is a very low level of community transmission, only minimal protocol changes may be necessary, and hyperventilation would seem safe in all patients without infectious symptoms or known COVID-19 contacts. For regions in which infection rates are very high, it may be reasonable to omit hyperventilation in certain clinical scenarios in which it is likely to be low yield, though at least in pediatric centers, I think this would be a small minority of patients. In the rare scenario that a patient with known or presumed COVID-19 required an EEG urgently, and was capable of performing hyperventilation, the EEG technologists should be wearing full personal protective equipment, but hyperventilation could be omitted as well. I agree with other authors’ recommendation that hyperventilation should never be performed with a mask.

The COVID-19 pandemic is a time of great uncertainty, and there is widespread fear and anxiety. Nevertheless, abandoning academic rigor when performing research and preparing clinical practice guidelines can only lead to more negative outcomes. I am particularly concerned about recommending changes to routine neurophysiology practices which would significantly decrease the diagnostic yield of tests when such changes are not supported by strong evidence, and there is no clear date or threshold as to when these changes would cease to be in effect. The “new normal” may be unavoidable but we still bear responsibility to ensure that we do not lower our level of clinical care unnecessarily.
